# Evidence for global cooling in the Late Cretaceous

**DOI:** 10.1038/ncomms5194

**Published:** 2014-06-17

**Authors:** Christian Linnert, Stuart A. Robinson, Jackie A. Lees, Paul R. Bown, Irene Pérez-Rodríguez, Maria Rose Petrizzo, Francesca Falzoni, Kate Littler, José Antonio Arz, Ernest E. Russell

**Affiliations:** 1 Department of Earth Sciences, University College London, Gower Street, London WC1E 6BT, UK; 2 Department of Earth Sciences, University of Oxford, South Parks Road, Oxford OX1 3AN, UK; 3 Departamento de Ciencias de la Tierra, Instituto de Investigación en Ciencias Ambientales (IUCA), Universidad de Zaragoza, Pedro Cerbuna, 12, E-50009 Zaragoza, Spain; 4 Dipartimento di Scienze della Terra “A. Desio”, Università degli Studi di Milano, via Mangiagalli 34, 20133 Milano, Italy; 5 Department of Geosciences, Mississippi State University, 108 Hilbun Hall, PO Box 5448, Oktibbeha, Mississippi 39762, USA; 6 Deceased

## Abstract

The Late Cretaceous ‘greenhouse’ world witnessed a transition from one of the warmest climates of the past 140 million years to cooler conditions, yet still without significant continental ice. Low-latitude sea surface temperature (SST) records are a vital piece of evidence required to unravel the cause of Late Cretaceous cooling, but high-quality data remain illusive. Here, using an organic geochemical palaeothermometer (TEX_86_), we present a record of SSTs for the Campanian–Maastrichtian interval (~83–66 Ma) from hemipelagic sediments deposited on the western North Atlantic shelf. Our record reveals that the North Atlantic at 35 °N was relatively warm in the earliest Campanian, with maximum SSTs of ~35 °C, but experienced significant cooling (~7 °C) after this to <~28 °C during the Maastrichtian. The overall stratigraphic trend is remarkably similar to records of high-latitude SSTs and bottom-water temperatures, suggesting that the cooling pattern was global rather than regional and, therefore, driven predominantly by declining atmospheric *p*CO_2_ levels.

## Abstract (web summary)

The Late Cretaceous experienced significant cooling, yet a lack of low-latitude records mean the regional extent of this cooling is poorly constrained. Linnert *et al.* present a TEX_86_ sea surface temperature record from a palaeolatitude of ~35 °N and show that Late Cretaceous cooling was global in nature.

## Graphical abstract

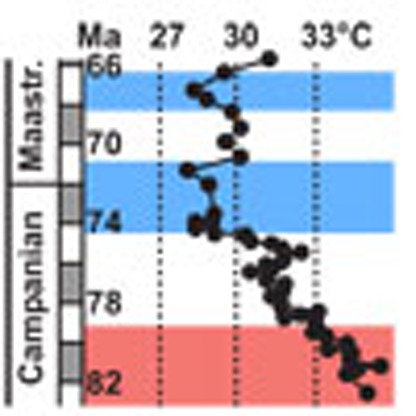

One of the warmest climates of the past 140 million years occurred in the early Late Cretaceous (late Cenomanian—early Turonian, between 95 and 90 Ma)^1^,^2^,^3^,^4^, with ice-free polar regions^5^, tropical sea surface temperatures (SSTs) greater than 35 °C (ref. 2) and shallow latitudinal temperature gradients^6^,^7^. The interval following this (late Turonian through Maastrichtian, ~90 to 66 Ma) is considered to have been a period of significant global cooling, possibly driven by a combination of declining *p*CO_2_ levels and opening ocean gateways^1^,^4^,^5^,^8^,^9^. Although general trends in Late Cretaceous climate evolution are relatively well established^1^,^4^,^5^, these inferences are largely based on either bulk fine-fraction carbonate or benthic foraminiferal stable isotope data, representing mixed (fine fraction) or bottom-water temperature records. The rate and structure of Late Cretaceous SST cooling is poorly constrained, as most reconstructions are limited to short, fragmentary and low-stratigraphic-resolution planktonic foraminifera δ^18^O records^6^,^7^,^9^,^10^,^11^. Furthermore, the recognition of early diagenetic recrystallization of planktonic foraminifera at the sea floor, or shortly after burial^12^, has led to the rejection of many estimates of Cretaceous low-latitude SSTs that were anomalously cool compared with equivalent modern latitudes (the so-called cool tropics paradox). The shortcomings of carbonate-based palaeothermometry can, in some cases, be circumnavigated by using organic palaeotemperature proxies, such as TEX_86_ (TetraEther indeX of tetraether consisting of 86 carbon atoms)^13^, which we apply here to a critical, but poorly quantified, interval of climate change—the Campanian–Maastrichtian.

The TEX_86_ palaeothermometer provides estimates of mean annual SST^13^, independent of initial seawater chemistry and, compared with carbonate microfossils, is less subject to the modifying effects of diagenesis^13^. The TEX_86_ proxy is based on the observed relationship between the ratio of different thaumarchaeotal membrane lipids (tetraether) and the mean annual temperature of the seawater in which the organisms lived^13^,^14^,^15^,^16^. However, TEX_86_ is still a relatively new method and development of the proxy is ongoing. For example, recent studies have suggested that TEX_86_ values may be influenced by archaeotal and thaumarchaeotal biology (taxonomy, diversity changes, physiology, life habitats)^15^,^16^,^17^ and oceanographic setting (for example, water depth), which has led to discussions about how best to apply the temperature calibration in studies of past climate^13^,^14^,^18^,^19^.

Our samples come from the Shuqualak–Evans borehole in Mississippi (USA) that comprises a ~240 m-thick sequence of shelfal, hemipelagic sediments. Deposition during the Cretaceous occurred at a palaeolatitude of ~35°N^20^, on a broad shelf bordering the subtropical western North Atlantic to the east and the proto-Caribbean region to the south (Fig. 1). Our age model is based on integrated calcareous nannofossil and planktonic foraminifera datums and indicates rapidly deposited Campanian sediments overlain by a Maastrichtian sequence with slower sedimentation rates (see Methods, Supplementary Table 1 and Supplementary Fig. 1 for a detailed age model description). The succession appears to be stratigraphically complete from the early to late Campanian, whereas the Santonian–Campanian transition and the overlying Maastrichtian sediments are relatively condensed. TEX_86_ analysis of the Shuqualak–Evans core reveals a significant cooling of ~7 °C during the Campanian, which we compare with records of SSTs from low and high latitudes, and a global compilation of bottom waters. The similarity in long-term trends in these datasets highlights the global nature of late Cretaceous cooling, whilst raising interesting questions regarding the sensitivities of past greenhouse climates to changing boundary conditions such as palaeogeography and atmospheric *p*CO_2_.

## Results

### TEX_86_ data and SST estimates

TEX_86_ values from the Shuqualak–Evans borehole decrease from a maximum of 0.90 in the lowermost Campanian to a minimum of 0.70 in the Campanian–Maastrichtian boundary interval (Fig. 2). In the lower Maastrichtian, the TEX_86_ values rise to 0.75, followed by a decrease to 0.71. In the upper Maastrichtian, values rise again to 0.78. The lower Campanian TEX_86_ values, in particular, are far higher than those observed in the modern ocean^14^, implying much higher SSTs in early Campanian times. Different calibrations exist between GDGT (Glycerol Dialkyl Glycerol Tetraether) relative abundances and SST (for example, TEX_86_, 1/TEX_86_, TEX_86_^H^, TEX_86_^L^, pTEX_86_, BAYSPAR)^14^,^19^,^21^. TEX_86_^H^ has been widely viewed as the most appropriate calibration for past greenhouse climates and is used here for discussion of our estimates of Cretaceous SSTs, as our measured values of TEX_86_ are high and the study site is a low-latitude setting^14^,^21^. However, the estimates we provide should be considered to be maximum values, as a recent study^18^ has suggested that TEX_86_^L^ might be more appropriate than TEX_86_^H^ at sites where the palaeowater depth was shallower than 1000, m, such as at Shuqualak. The recently developed BAYSPAR method^19^ yields similar average values to TEX_86_^H^, although the uncertainty estimate suggests a wide range of possible values around the average (±~5 to ~8 °C), due to issues related to the regression parameters and extrapolation of the TEX_86_ proxy^19^. For completeness, we give SSTs calculated using TEX_86_^L^ and BAYSPAR in the Supplementary Table 2 and Supplementary Fig. 2 (see Methods). Although the choice of calibration does change the absolute temperature estimates from the Shuqualak–Evans borehole, critically the stratigraphic trends remain the same (Supplementary Fig. 2). The TEX_86_^H^ calibration indicates maximum SSTs of ~34–36 °C for the earliest Campanian and ~28–29 °C for the Campanian–Maastrichtian transition (Fig. 2). The Maastrichtian SSTs vary between 28° and 31 °C, showing some evidence for warming and cooling events, but these variations are within the statistical error of the TEX_86_^H^ calibration (±2.5 °C)^14^, and the magnitude of change in the Maastrichtian should be interpreted with caution.

## Discussion

The maximum TEX_86_^H^-based SST estimates of 28–35 °C from the Shuqualak–Evans borehole suggest that Late Cretaceous climate was consistently warmer at ~35 °N than at present (average SSTs at 35 °N in the modern ocean are ~20 °C), and this conclusion is broadly true even using the TEX_86_^L^ calibration (Supplementary Fig. 2). These estimates of low-latitude SST are consistent with recently published TEX_86_ data from Israel^22^, which suggest a range of SSTs from ~23 to 33 °C (using TEX_86_^H^) during the Campanian–Maastrichtian at a palaeolatitude of 5° to 15 °N. Compared with the data from Shuqualak–Evans, the data from Israel display a greater range of SSTs and lower maximum values, which may be the result of deposition within an upwelling system. In contrast to these TEX_86_-based estimates of SSTs, a previous study based on δ^18^O palaeothermometry of mixed-layer-dwelling planktonic foraminifera from Blake Nose (western North Atlantic ~30 °N palaeolatitude) suggested SSTs of 19–25 °C^7^, which are equal to, or cooler than, present day SSTs at the equivalent latitude. It has been suggested that early diagenetic recrystallization of planktonic foraminifera could account for some of the lower temperatures reported from the Coniacian–Maastrichtian of Blake Nose^7^. Comparison with the TEX_86_ data (using either TEX_86_^H^, TEX_86_^L^ or BAYSPAR) from Shuqualak–Evans lends some support to this explanation for the mid to late Campanian (for example, *R. calcarata* zone). Furthermore, the highest SSTs we reconstruct (35 °C using TEX_86_^H^) are broadly consistent with (sub)tropical temperature estimates from other Mesozoic and Paleogene greenhouse intervals. For example, low- to mid-latitude mid-Cretaceous (Cenomanian–Turonian) and early Paleogene (Eocene) data from exceptionally well-preserved foraminiferal δ^18^O and TEX_86_ suggest that SSTs of 33 °C and above were typical in these greenhouse climate regimes^23^,^24^,^25^.

The addition of our new data to existing high-quality Late Cretaceous SST records from the equatorial^2^,^24^ and North Atlantic^26^, South Atlantic^6^,^7^, Tanzania^12^,^25^ and tropical Pacific^27^ provides an overview of the spatial and temporal evolution of SSTs for this time interval (Fig. 3 and Supplementary Fig. 3). Although limited by the number of available records, this compilation suggests a very shallow latitudinal temperature gradient during the Turonian, which steepened in the Campanian through Maastrichtian. Similar SSTs at 0° and 35°N in the North Atlantic during the Turonian–earliest Campanian may be due, in part, to the relative isolation and restriction of the basin, which was only connected by shallow and/or restricted gateways to the South Atlantic, Pacific and Tethys oceans at that time. However, the similarity between North Atlantic SSTs and estimates from the southern Tethys^22^ may suggest that North Atlantic temperatures were not remarkably different from other ocean basins at low latitudes, although, clearly, more good-quality data from the Tethys and Pacific are required to fully validate this hypothesis.

The similarity between Campanian–Maastrichtian low- (this study) and high-latitude^6^,^7^ SST trends and the global benthic foraminiferal δ^18^O record during the Campanian–Maastrichtian^4^,^5^ indicates that the Campanian cooling, evident in all datasets, was not solely a high-latitude phenomenon, but represents a global event. This cooling coincided with, and may have been related to, reconfiguration of oceanic gateways^28^,^29^ and hence deep, intermediate and shallow ocean circulation^4^,^29^,^30^,^31^. However, for significant deep- and surface-water cooling to occur across a wide range of latitudes, in both upwelling and non-upwelling settings, we suggest that declining atmospheric *p*CO_2_ levels^32^, possibly due to decreasing ocean crust production^33^, were the ultimate driver of this long-term climate evolution. The changing tectonic configuration may have led to slight differences in the timing and pattern of change in different regions and water depths, a hypothesis that could be tested with improved age-models for all critical sites and improved estimates of the timing of key tectonic events. The steepening of latitudinal temperature gradients during the Late Cretaceous is consistent with predictions from climate modelling^34^ that suggest that the latitudinal gradient is strongly dependent on *p*CO_2_ levels, with shallower gradients at higher *p*CO_2_. Short-term variability in Maastrichtian benthic foraminifera δ^18^O has previously been interpreted as representing repeated reversals of deep-ocean circulation from low- to high-latitude sources^10^. However, the existence of broadly synchronous trends in our SST data, and temperature-indicative changes in calcareous nannofossil assemblages^8^, suggest that the benthic δ^18^O record may also be responding, ultimately, to changes in climate, possibly driven by *p*CO_2_.

Our new data provide a critical addition to the understanding of climate evolution, from extreme warmth during the mid-Cretaceous to the termination of greenhouse conditions at the end of the Eocene. The data demonstrate that the transition from the so-called ‘supergreenhouse’ conditions of the mid-Cretaceous (Aptian–Turonian) to the cooler greenhouse world of the later Cretaceous and early Paleogene, occurred through gradual global cooling, rather than rapid, stepped changes, and that cooling was not confined to high latitudes. A similar transition has also been documented for the Eocene, before the switch to icehouse-mode climates in the Oligocene, albeit with much lower magnitude cooling at low latitudes^23^,^35^,^36^. The long-term cooling trend at high latitudes in the Eocene was likely caused by the opening of the Tasman Gateway during the early to middle Eocene, rather than a simple decrease in atmospheric *p*CO_2_ alone, which would have led to more substantial cooling of (sub)equatorial surface waters than is observed^36^. It has been suggested that, during the late Eocene, declining *p*CO_2_ levels eventually crossed a critical threshold (of ~750 p.p.m.) at the Eocene/Oligocene (E/O) boundary, which allowed the rapid growth of the Antarctic ice sheet and a stepped climate change into an icehouse state^37^,^38^,^39^. Why, then, did significant continental ice-sheet growth occur at the E/O boundary, but not during the late Cretaceous, given the cooler Late Campanian and Maastrichtian water temperatures (surface and deep) and cooling at both high and subequatorial latitudes^5^? Proxy reconstructions and models do not provide an unambiguous picture of the temporal evolution of Late Cretaceous–early Paleogene atmospheric *p*CO_2_ levels and trends^40^, although, overall, the latest Cretaceous appears to have been characterized by lower *p*CO_2_ levels than the mid-Cretaceous. It is therefore unclear whether the absence of continental scale glaciation was because *p*CO_2_ did not fall sufficiently far, or because the *p*CO_2_ threshold limit for ice-sheet growth was lower during the Late Cretaceous compared with the Eocene, due to different baseline conditions, such as ocean gateway configurations. Modelling of the E/O boundary event^37^ suggests that open tectonic gateways around Antarctica are not necessarily required for initiation of continental scale glaciation, but they can exert a control on the amount of *p*CO_2_ decline needed for glaciation. In the case of the E/O boundary, modelling suggests that, for major glaciation to occur with a closed Drake Passage, the *p*CO_2_ threshold would be ~140 p.p.m. lower than in a scenario with the Drake Passage open. During the Late Cretaceous, both the Drake Passage and the Tasman Gateway were closed and, thus, it is likely that the threshold for glaciation was <~600 p.p.m.V. Understanding why a major ice sheet was not initiated in the Late Cretaceous during an interval of marked global cooling, given some superficial similarities to Eocene climate trends and absolute values, is an intriguing challenge, which, if addressed through additional data and modelling, could provide valuable insights into the long-term controls on cryosphere development during greenhouse and ‘doubthouse’ conditions, climate sensitivity to changing *p*CO_2_, and the plausibility of glacioeustatic sea-level change during the Late Cretaceous and Early Paleogene.

## Methods

### Core location and palaeogeography

The Shuqualak–Evans core is from Shuqualak, Mississippi, USA (32°58′49′′N, 88°34′8′′W) and was sampled for TEX_86_ from a depth of 9.45 m down to 251.46 m, spanning the Santonian/Campanian boundary interval through to the uppermost Maastrichtian.

**Figure 1:**
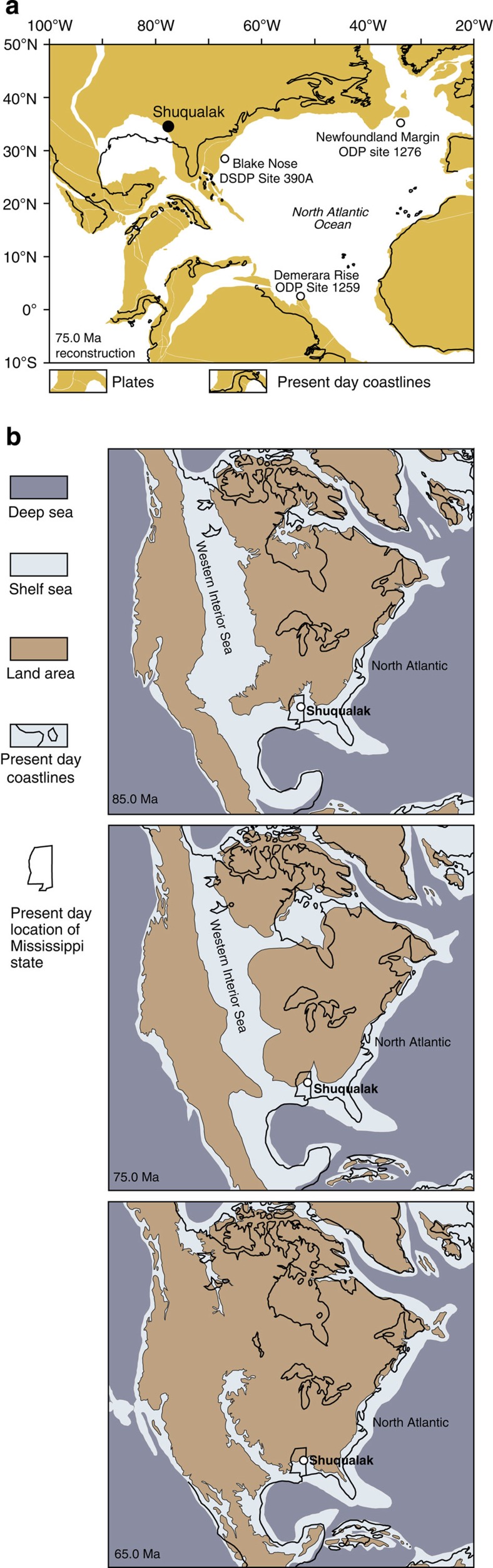
Latest Cretaceous palaeogeography of the North Atlantic. (**a**) Simplified late Campanian (75 Ma) plate tectonic reconstruction adapted from the Ocean Drilling Stratigraphic Network (ODSN) palaeomap project ( http://www.odsn.de/odsn/services/paleomap/paleomap.html). The location of the Shuqualak–Evans borehole sampled in this study is shown as a black circle; the locations of DSDP/ODP sites discussed in the text are shown as small open circles. (**b**) North American Palaeogeography for the Late Santonian (85.0 Ma)–Early Palaeocene (65.0 Ma) interval. The location of Shuqualak is indicated as an open circle within the outline of Mississippi. Palaeogeographic maps are drawn after the original maps (65, 75, 85 Ma) of Ron Blakey, NAU Geology ( http://jan.ucc.nau.edu/%7Ercb7/nam.html).

**Figure 2:**
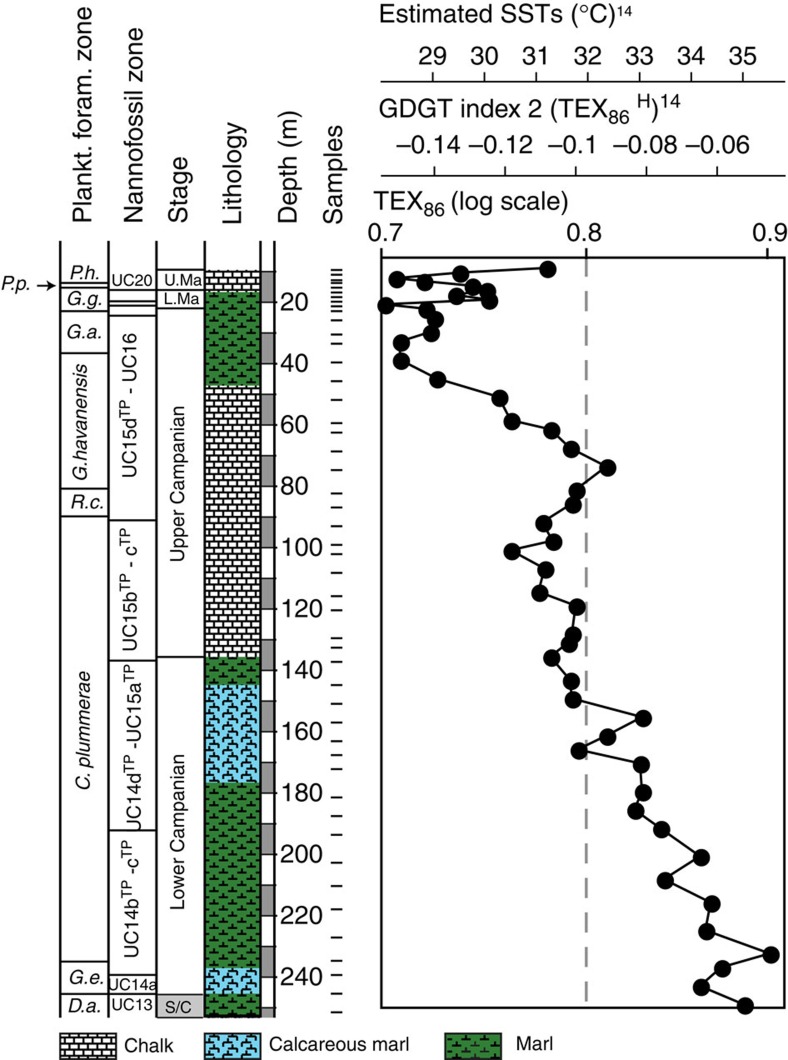
TEX_86_ data and calculated SSTs from the Shuqualak–Evans borehole. Palaeo-SST estimates are based on the TEX_86_^H^ calibration^14^. Note that TEX_86_ is plotted on a logarithmic scale. The *Abathomphalus mayaroensis* and *Racemiguembilina fructicosa* Planktonic Foram Zones cannot be assigned in the Shuqualak–Evans core: *A. mayaroensis* has not been recorded, probably due to environmental and/or palaeogeographical constraints and base *R. fructicosa* is recorded in the same horizon as base *Pseudoguembelina hariaensis*, likely due to the very low Maastrichtian sedimentation rate and not because of a hiatus, since all the nannofossil zones are present. *C. plummerae*, *Contusotruncana plummerae; D. a.*, *Dicarinella asymetrica; G. a*., *Globotruncana aegyptiaca; G. e.*, *Globotruncanita elevate*; *G. g.*, *Gansserina gansseri; G. havanensis*, *Globotruncanella havanensis;* L. Ma., Lower Maastrichtian; *P. h.*, *Pseudoguembelina hariaensis; P. p.*, *Pseudoguembelina palpebra*; *R. c.*, *Radotruncana calcarata;* S/C, Santonian–Campanian boundary interval; U. Ma., Upper Maastrichtian.

Figure 1 shows a model of the palaeogeographic evolution of North America during the latest Cretaceous. In these reconstructions and others (for example, ref. 20) the Shuqualak–Evans borehole was situated on a broad shelf bordering the North Atlantic Ocean and Gulf of Mexico during the latest Cretaceous. Surface ocean circulation reconstructions (summarized in ref. 31) suggest that this location was likely not influenced by waters of the Western Interior Seaway and this is supported by calcareous nannofossil assemblage components and abundances, which suggest an open ocean water mass.

### Biostratigraphy and age-model

The age-model for the Shuqualak–Evans core is based on integrated calcareous nannofossil^41^ and planktonic foraminifera^42^ biostratigraphic datums (Supplementary Fig. 1 and Supplementary Table 1), with calibrated absolute ages taken from Gradstein *et al.*^42^ For the uppermost Campanian through Maastrichtian (25.91–9.45 m), the age model is calculated as a linear function, with tie points at base *Lithraphidites quadratus* (16.76 m, 69.18 Ma^42^) and base *Micula prinsii* (12.80 m, 67.30 Ma^42^). This suggests a low sedimentation rate of ~2.1 m per Myr ago. We assign a minimum age of 66 Ma to the shallowest sample (9.45 m), as the nannofossils indicate a Cretaceous age yet the age-model predicts a Palaeocene age and we cannot further refine the age-model above 12.80 m, which appears to be topmost Maastrichtian. For most of the Campanian (245.36–30.48 m), the age-model is a linear function, with tie points at base *Broinsonia parca* subsp. *constricta* (239.27 m, 81.38 Ma^42^) and base *Uniplanarius sissinghii* (134.11 m, 77.61 Ma^42^). This indicates a high sedimentation rate of ~27.9 m per Myr. For the lowermost sample analysed for geochemistry, the age model is constrained by base *Broinsonia parca* subsp. *parca* (245.36 m, 81.43 Ma^42^) and the presence of *Arkhangelskiella cymbiformis* (252.83 m, assigned maximum age of 83.20 Ma^42^), indicating a low sedimentation rate of ~4.2 m per Myr ago. Note that the co-occurrence of *Dicarinella asymetrica* and *A. cymbiformis* (at 251.46 m) suggests that the age of the lowermost sample analysed for TEX_86_ is around the Santonian/Campanian boundary interval. The TEX_86_ and SST data are given in relation to the sample depths in Supplementary Fig. 2 and Supplementary Table 2.

### GDGT extraction and analysis

Samples were solvent extracted using the technique previously published by Schouten *et al.*^13^,^43^ Approximately 6 g powdered sample was ultrasonically extracted using one time methanol, three times dichloromethane (DCM)/methanol (1:1, v/v) and three times DCM. All extracts were combined and dried under a continuous N_2_ flow at 40 °C. Any water remaining in the samples was removed by passing the extracts (dissolved in DCM/methanol (3:1, v/v)) over a column containing anhydrous Na_2_SO_4_. Extracts were split into polar and apolar fractions by column chromatography, using hexane/DCM (9:1, v/v) and DCM/methanol (1:1, v/v) sequentially as the eluents and Al_2_O_3_ as the stationary phase. The polar extract containing the targeted GDGTs was dissolved in hexane/propanol (99:1, v/v) and then filtered through a PTFE (polytetrafluoroethylene) 0.45 μm filter. After drying down, the samples were redissolved in a certain volume of hexane/propanol (99:1, v/v), which depends on the weight of each polar fraction. All 48 samples were analysed in the Department of Earth Sciences at UCL on an Agilent 1200 series HPLC attached to a G6130A single-quadrupole mass spectrometer. The analytical protocol followed is as described in Schouten *et al.*^43^. The abundance of both isoprenoid and branched GDGTs was measured in selective ion monitoring mode. Ion peaks of the respective GDGTs were integrated to determine the relative abundance of each molecule in the sample. These abundances were then used to determine the TEX_86,_ TEX_86_^H^ (GDGT-index 2), and TEX_86_^L^ (GDGT-index 1) indices^13^,^14^. These indices are defined as follows:


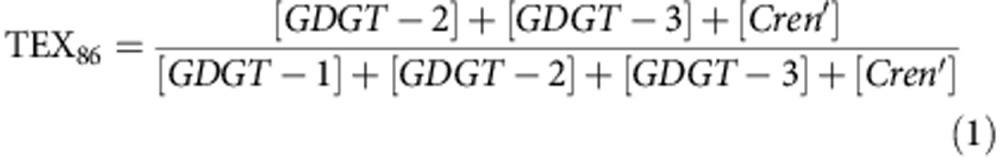



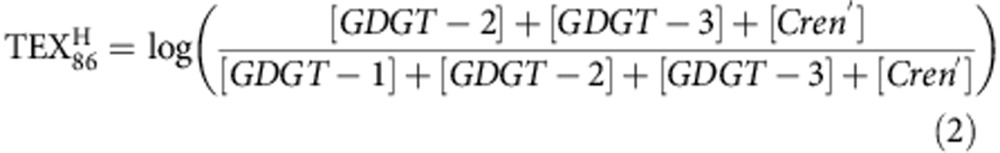



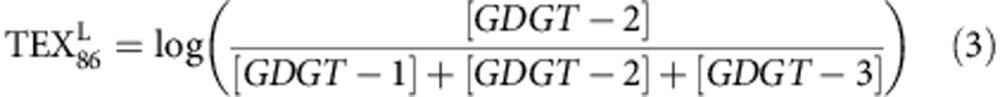


where Cren' represents the crenarchaeol regioisomer. Our calculated TEX_86,_ TEX_86_^H^ (GDGT-index 2) and TEX_86_^L^ (GDGT-index 1) values are presented in Supplementary Table 2 and Supplementary Fig. 2.

### SST calculations from GDGT abundances

To calculate SSTs, we used the following equations from Kim *et al.*^14^ which are based upon a comprehensive modern core-top dataset:


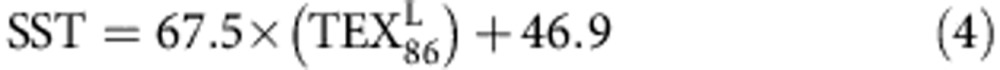



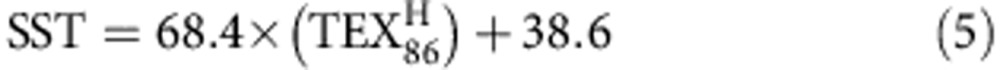


**Figure 3:**
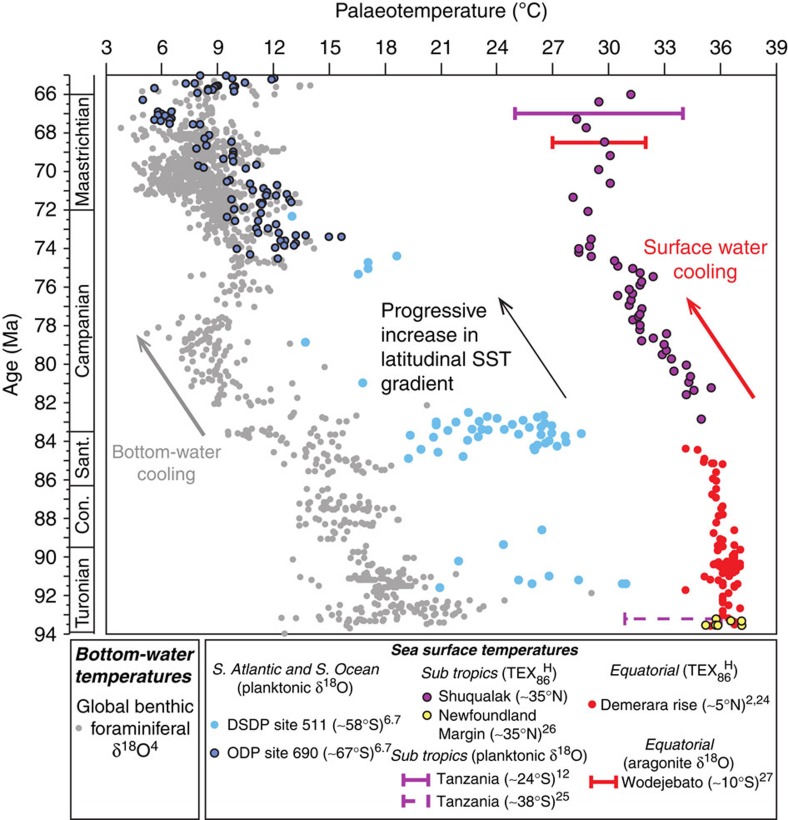
Comparison of key Late Cretaceous bottom water and sea surface temperature records. Benthic and planktonic foraminiferal estimates of temperature have been recalculated using the δ^18^O data ofrefs 6,7,12,25 (see Methods for details). Note the Tanzanian planktonic foraminifera^12^,^25^ have not been sorted by depth ecology, and consequently the range of SSTs calculated likely encompasses estimates from mixed-layer to thermocline-dwelling species. It is likely that the warmest temperatures are most representative of mixed-layer conditions. SST estimates from metastable carbonates^27^ have been taken directly from the literature, but note that these estimates are minimum values, based on conservative assumptions of *δ*_w_. SST estimates from published TEX_86_ data^2^,^24^,^26^ have been recalculated where necessary, using the TEX_86_^H^ proxy. The calibration error associated with TEX_86_^H^ is ±2.5 °C^14^. The Turonian-age data from the Newfoundland margin^26^ only include data from after Oceanic Anoxic Event 2. Published age-models have been used throughout.

At temperatures >15 °C expected during greenhouse periods in Earth history, it has been recommended^14^ that the TEX_86_^H^ index should be used, as both indices should yield the same estimate of SST according to the modern core-top calibration dataset, but TEX_86_^H^ has an associated calibration error that is significantly lower (±2.5 °C) compared with that of TEX_86_^L^ (±4 °C). However, application of TEX_86_^L^ and TEX_86_^H^ to datasets from Early Cenozoic sediments^21^ has revealed that they do not always yield the same estimates of SST, with TEX_86_^L^ generally yielding lower temperatures. At high-latitude Palaeocene–Eocene sites in New Zealand, Hollis *et al.*^21^ found that TEX_86_^L^ yielded SST estimates more comparable to inorganic proxies (δ^18^O, Mg/Ca) than TEX_86_^H^, but suggested that, at low latitudes and high TEX_86_ values, TEX_86_^H^ might be as appropriate. In their analysis of the ability of the different GDGT-based proxies to replicate inorganic SST estimates, Hollis *et al.*^21^ noted that, at high TEX_86_ values above 0.70, the over-estimation of SST is less than 5 °C using TEX_86_^H^. Furthermore, at TEX_86_ values above 0.75, they suggested that TEX_86_^L^ underestimates SST. The oxygen-isotopic compositions of well-preserved planktonic foraminifera of Cenomanian–Santonian age from Demerara Rise (~5 °N palaeolatitude) yield SSTs typically in the range of 35 to >37 °C^24^, comparable to SSTs derived by TEX_86_^H^ of 35 to 37 °C from the same site (recalculated by us from the published TEX_86_ data). Therefore, through the consideration of previous suggestions and multiproxy records of low-latitude mid-Cretaceous SSTs, it would initially appear that the TEX_86_^H^ calibration represents the best estimate of SSTs for the Shuqualak–Evans borehole. Furthermore, in order to be able to compare our new data with previously published Late Cretaceous TEX_86_ data^2^,^24^, from which TEX_86_^L^ values are not yet available, we have had to use the TEX_86_^H^ index in Fig. 3. Nonetheless, in Supplementary Figs 2 and 3 we show both the TEX_86_^H^ and TEX_86_^L^ data (where possible) and the calculated SSTs to illustrate the potential range of values. We also present in Supplementary Fig. 2 SSTs calculated using the recently developed BAYSPAR approach^19^. The BAYSPAR model considers how the relationship between TEX_86_ and temperature varies spatially and considers uncertainties in the modern SST-TEX_86_ relationship. Critically, the stratigraphic trends for Shuqualak–Evans are near identical, irrespective of which proxy is used and, thus, our conclusions regarding the temporal evolution of the direction of Late Cretaceous climatic and latitudinal gradient change remains valid, even if absolute values are harder to constrain.

Recent work, based on an analysis of modern water-column GDGT abundance profiles, the core-top calibration dataset and a compiled Paleogene dataset, suggests that TEX_86_^H^ may be less appropriate than TEX_86_^L^ for use at sites where the water depth was approximately shallower than 1000, m (such as Shuqualak). This is likely due to variations in the export dynamics of individual GDGT compounds with depth, and an apparent temperature/water depth bias in the core-top calibration dataset^18^. In both the modern core-top calibration dataset and the Paleogene dataset, sites deposited in <1,000 m of water exhibit low GDGT-2/GDGT-3 ([2]/[3]) ratios and high offsets between SSTs calculated by TEX_86_^H^ and TEX_86_^L^ (ΔH-L). We have been able to obtain the raw GDGT data for the sites on Demerara Rise, which were thought to have been deposited at water depths of <1500, m^44^. The GDGTs from these sites exhibit low [2]/[3] ratios and high ΔH-L, which Taylor *et al.*^18^ suggest is characteristic of water depths <1,000 m. We therefore contend that it is appropriate in Supplementary Fig. 3 to compare Demerara Rise and Shuqualak–Evans using either TEX_86_^H^ or TEX_86_^L^ (as the water depth of all sites was likely about, or shallower than, 1000, m). The application of the TEX_86_^L^ calibration to our data from Shuqualak–Evans suggests SSTs of ~28 °C for the earliest Campanian and ~20 °C for the Campanian–Maastrichtian transition, which are some ~7–8 °C lower than the estimates of the TEX_86_^H^ model, and much closer to modern SSTs at comparable latitudes, which perhaps is surprising given that Late Cretaceous *p*CO_2_ levels are thought to have been higher than present (~600 to 800 p.p.m. versus 280 p.p.m. for the preindustrial modern)^40^. Furthermore, the use of TEX_86_^L^ suggests almost no temperature gradient between 5° and 60° absolute palaeolatitude during the Turonian–Santonian, which also seems unlikely. The issue of how best to calculate temperatures from GDGT data is ongoing and it may be that a calibration based on suspended particulate organic matter may overcome some of the issues described above^16^,^18^.

Repeated analysis of an in-house standard and selected samples suggest that analytical reproducibility of TEX_86_ is better than ±0.009, in line with previous studies^45^ that suggest an analytical error for TEX_86_ index of ±0.01. In our data, we estimate that the error on SST estimates associated with analytical precision is <±0.4 °C for the TEX_86_^H^ calibration, and <±0.6 °C using TEX_86_^L^. Analytical error is far less than the standard error associated with the core-top calibrations, which for TEX_86_^H^ is ±2.5 °C, and for the TEX_86_^L^ calibration is ±4.0 °C^21^.

### BIT and MI indices

The GDGTs analysed for the TEX_86_ palaeotemperature proxy are mainly produced by marine thaumarchaeota. However, the same GDGTs are also produced by terrestrial soil organisms and methanotrophs.

GDGTs of terrestrial origin can be washed into the marine realm by rivers, potentially biasing SST reconstructions. Apart from isoprenoid GDGTs, which are used for the TEX_86_ techniques, terrestrial organisms also produce branched GDGTs^46^. Branched GDGTs are typical of terrestrial organisms, but they do not occur among marine thaumarchaeota^46^. Thus, the branched GDGTs are used to quantify the terrestrial GDGT contamination in marine sediment samples by calculating the Branched and Isoprenoid Tetraether (BIT) index^46^. The BIT index is based on the ratio of branched GDGTs to the isoprenoid GDGT crenarchaeol^46^. In our study, the measurement of these branched GDGTs was included in the analytical protocol. There is no significant relationship between BIT and TEX_86_ in our data (Supplementary Fig. 4) and the BIT index is between 0.05 and 0.15 (Supplementary Fig. 2). This is well below the recommended threshold of 0.2, above which soil microbial contamination may be problematic^47^. Thus the SST estimates made in this study are probably not altered by an influence of terrestrial GDGTs.

GDGTs produced by methanotrophs within the sediments can distort the TEX_86_ signal and lead to erroneous estimates of palaeotemperature. The degree of influence of methanotropic archaea can be estimated using the Methane Index (MI)^17^. Normal marine sediments have values <0.3, whereas sediments influenced by high rates of methane production have values >0.5. The interval from 0.3 to 0.5 marks the transition between the two environments^17^. The MI values from Shuqualak vary from ~0.1 to 0.25 (Supplementary Fig. 2), suggesting normal marine conditions and a lack of GDGT production by methanotrophs. We therefore conclude that methanotrophic production of GDGTs has not impacted adversely on our TEX_86_ records.

### Palaeotemperatures from foraminiferal oxygen isotopes

In Fig. 3 and Supplementary Fig. 3, we show estimates of palaeotemperature based upon the oxygen-isotopic (δ^18^O) composition of a global compilation of benthic foraminiferal data^4^, selected low-latitude planktonic foraminiferal datasets^12^,^25^ and mixed-layer-dwelling planktonic foraminifera from southern high-latitude sites (DSDP Site 511 and ODP Site 690)^6^,^7^. Calcareous dinoflagellate cysts from ODP Site 690 have similar d18O values to planktic foraminifera^48^ but are not included as a suitable temperature calibration for Cretaceous dinoflagellates is not available. Note for the Turonian-age samples from Tanzania, we have recalculated the SST range using the typical range of planktonic δ^18^O values measured (−4 to −5 ‰)^25^. In order to apply a consistent approach to calculating temperatures from δ^18^O of foraminiferal calcite, we have recalculated temperatures from the original published oxygen-isotopic datasets. We used equation (6) to calculate temperatures^49^:


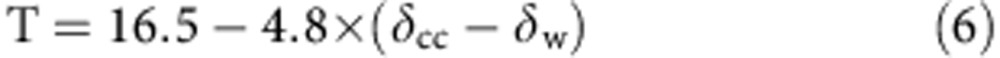


where T=temperature (°C), *δ*_cc_=δ^18^O of foraminiferal calcite (‰, VPDB) and *δ*_w_=δ^18^O of ambient seawater.

We used a *δ*_w_ value of −1.27, which includes a correction of −0.27 for the conversion of Vienna Standard Mean Ocean Water to Vienna PeeDee Belemnite and an ice volume value of −1‰. For calculation of SSTs from planktonic foraminifera, we applied an additional latitudinal salinity correction^50^ to *δ*_w_, using the palaeolatitudes of DSDP Site 511 and ODP Site 690 in the Late Cretaceous (58°S and 67°S, respectively) and equation (7):


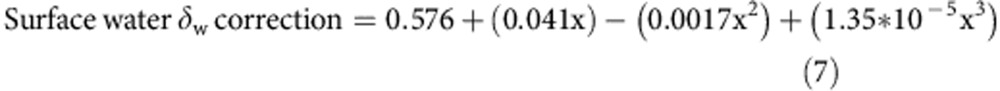


where x=absolute palaeolatitude between 0° and 70°. Palaeolatitudinal positions for each site were using the ODSN Plate reconstruction software ( http://www.odsn.de/odsn/services/paleomap/paleomap.html).

## Author contributions

J.A.L., S.A.R. and P.R.B. conceived the project. Access to the core was provided by E.E.R. C.L., K.L. and S.A.R. generated the geochemical data. J.A.L., I.P.-R., M.R.P., F.F. and J.A.A. generated the biostratigraphic data and P.R.B., J.A.L. and C.L. produced the age model. C.L., S.A.R., P.R.B. and J.A.L. drafted the manuscript and figures. All authors commented on the manuscript.

## Additional information

**How to cite this article:** Christian, L. *et al.* Evidence for global cooling in the Late Cretaceous. *Nat. Commun.* 5:4194 doi: 10.1038/ncomms5194 (2014).

## Supplementary Material

Supplementary Information Supplementary Figures 1-4 and Supplementary Tables 1-2
