# Supplementary material for: Evidence for global cooling in the Late Cretaceous
Source: Nat Commun. 2014 Jun 17;5:4194. doi: 10.1038/ncomms5194 (PMC4082635; doi:10.1038/ncomms5194)
Supplement: Supplementary Information — Supplementary Figures 1-4 and Supplementary Tables 1-2 [file ncomms5194-s1.pdf]

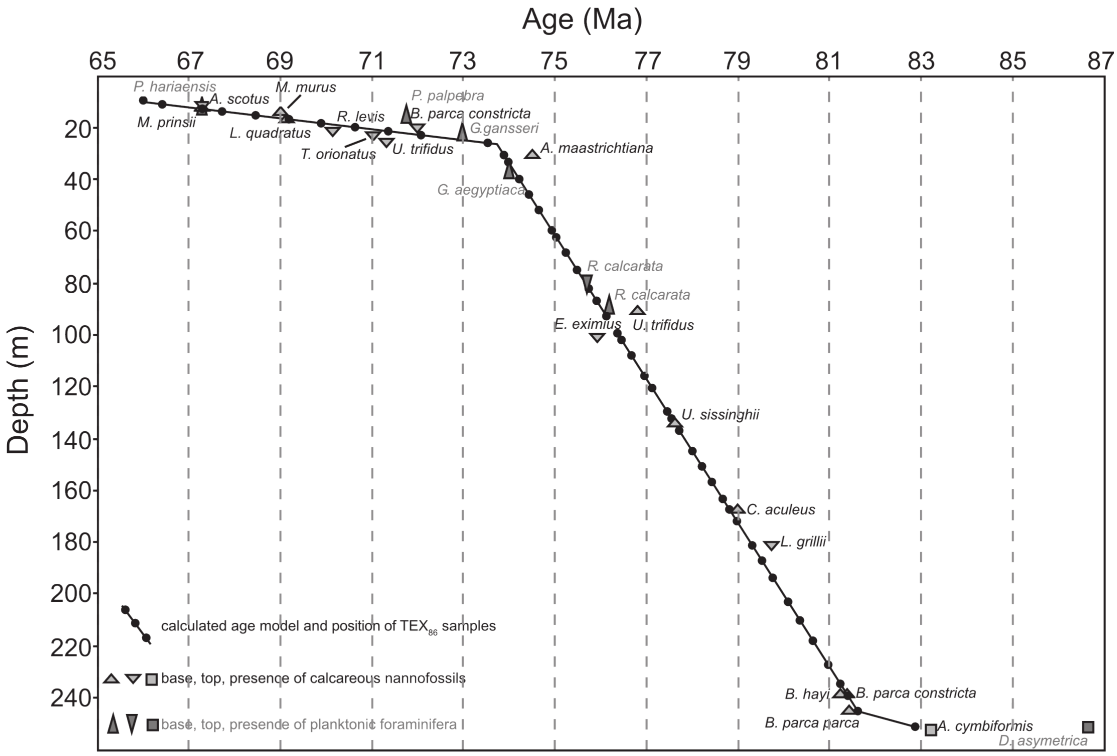

Supplementary Figure 1: Depth/Age plot of the Shuqualak-Evans core with major bioevents. Bioevents are given as bases (marking the evolutionary appearance of a species) and tops (marking the extinction of a species). The age-model is marked by a solid line connecting the three calculated linear functions (function1: 9.45m - 25.91m, function2: 30.48m - 245.36m, function3: 245.36m - 252.83m).

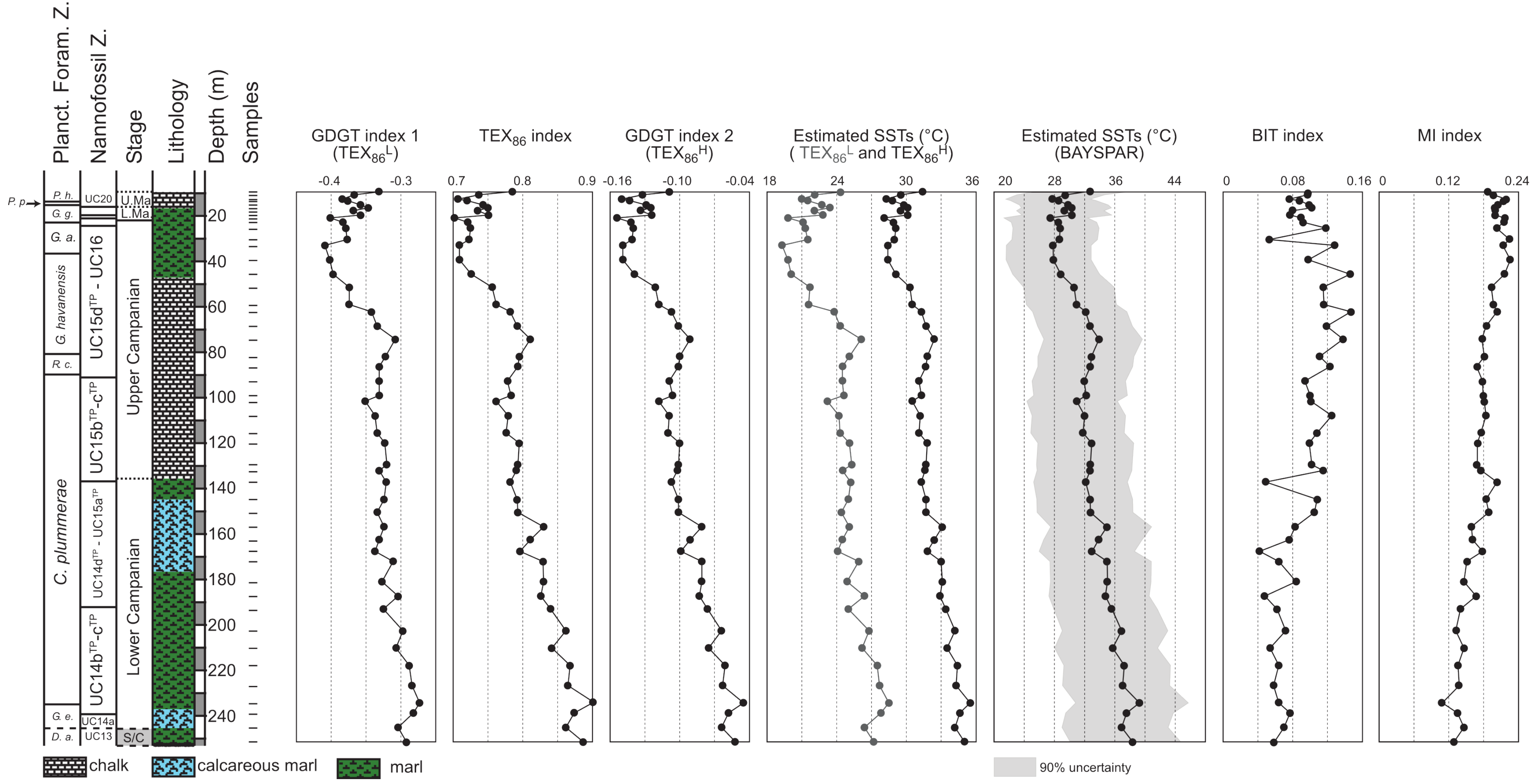

Planct. Foram. Z.
Nannofossil Z.
Stage
Lithology
Depth (m)
Samples
GDGT index 1 (TEX_{86}^{L})
TEX_{86} index
GDGT index 2 (TEX_{86}^{H})
Estimated SSTs (°C) (TEX_{86}^{L} and TEX_{86}^{H})
Estimated SSTs (°C) (BAYSPAR)
BIT index
MI index
P. p.
P. h.
G. g.
G. a.
G. havanensis
R. c.
C. plummerae
G. e.
D. a.
UC20
UC15d^{TP} - UC16
UC15b^{TP}-c^{TP}
UC14d^{TP} - UC15a^{TP}
UC14b^{TP}-c^{TP}
UC14a
UC13
U.Ma
L.Ma
Upper Campanian
Lower Campanian
S/C
chalk
calcareous marl
marl
90% uncertainty

last page:

Supplementary Figure 2: Palaeoclimatic interpretations of TEX_{86} data and other GDGT-based indexes . TEX_{86} and TEX_{86}^{H} after Eqs 1 and 2; TEX_{86}^{L} after Eq. 3; TEX_{86}^{L} temperature and TEX_{86}^{H} temperature after Eqs 4 and 5. BAYSPAR temperature (Prior Mean: 30, Prior STD: 6) after reference 19. Branched and Isoprenoid Tetraether (BIT) Index after reference 46. Methane Index (MI) after reference 17. The *Abathomphalus mayaroensis* and *Racemiguembilina fructicosa* Planktonic Foram Zones cannot be assigned in the Shuqualak-Evans core: *A. mayaroensis* has not been recorded, probably due to environmental and/or palaeogeographical constraints and base *R. fructicosa* is recorded in the same horizon as base *Pseudoguembelina hariaensis*, likely due to the very low Maastrichtian sedimentation rate and not because of a hiatus, since all the nannofossil zones are present.Abbreviations: see caption for Figure 2.

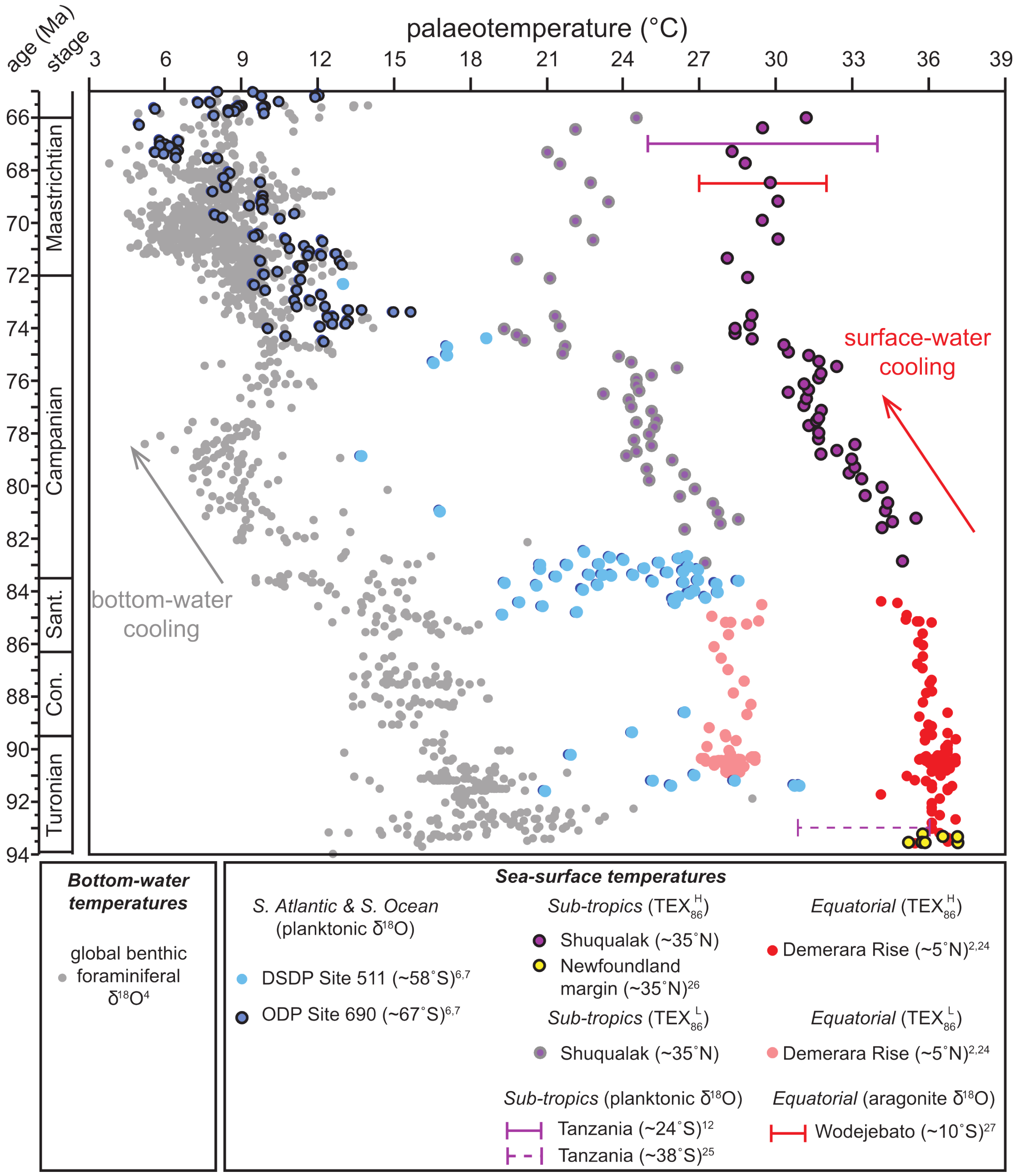

Supplementary Figure 3: Comparison of key Late Cretaceous bottom-water and sea-surface temperature records. Benthic and planktonic foraminiferal estimates of temperature have been recalculated using the $\delta^{18}O$ data of refs 6, 7, 12, 25 (see Supplementary Information for details). Note the Tanzanian planktonic foraminifera[12,25] have not been sorted by depth ecology and, consequently, the range of SSTs calculated likely encompasses estimates from mixed-layer- to thermocline-dwelling species. It is likely that the warmest temperatures are most representative of

mixed-layer conditions. SST estimates from metastable carbonates[27] have been taken directly from the literature, but note that these estimates are minimum values, based on conservative assumptions of $\delta^{18}O$. SST estimates from published TEX_{86} data[2,24,26] have been recalculated, where necessary, using the TEX_{86}^{H} proxy, and, where possible, TEX_{86}^{L} (GDGT abundance data was not available for all samples). The calibration error associated with TEX_{86}^{H} is ±2.5˚C[14] and ±4.0˚C with TEX_{86}^{L}. The Turonian-age TEX_{86}^{H} data from the Newfoundland margin[26] only include data from after Oceanic Anoxic Event 2. Published age-models have been used throughout.

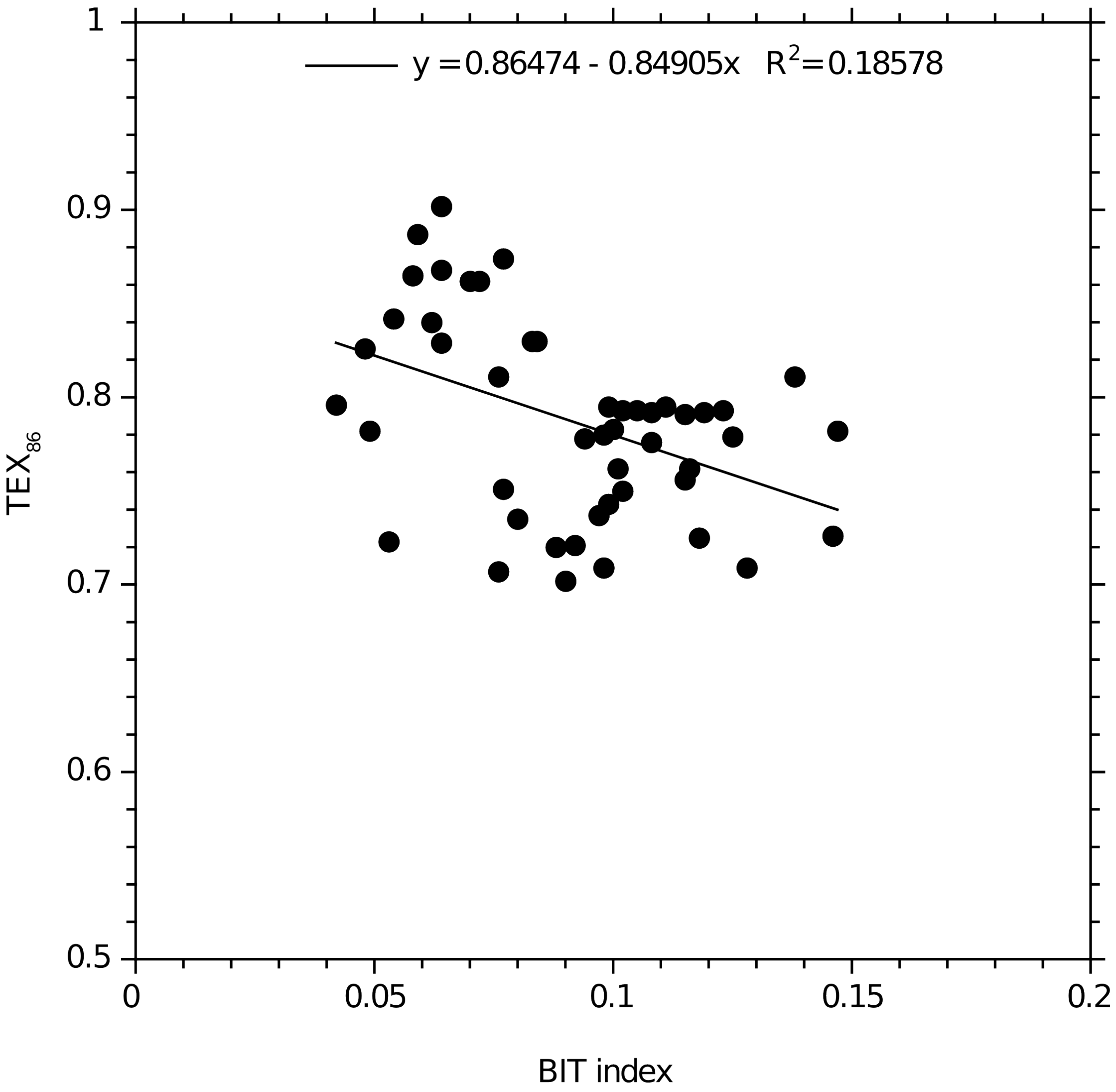

Supplementary Figure 4: Cross-plot of TEX_{86} values against BIT index.

Supplementary Table 1: Table of bioevents. Depths and bioevents marked in bold were used to construct the age-model. N = nannofossil, F = planktonic foraminifera. Ages from ref. 42.

Depth (m)	Bioevents (type, age)
**12.80**	**base *Micula prinsii* (N, 67.30Ma),** base *Pseudoguembelina hariaensis* (F, 67.30Ma), top *Acuturris scotus* (N, 67.30Ma)
13.72	base *Micula murus* (N, 69.00Ma)
15.24	base *Pseudoguembelina palpebra* (F, 71.75Ma)
**16.76**	**base *Lithraphidites quadratus* (N, 69.18Ma)**
19.81	top *Broinsonia parca* subsp. *constricta* (N, 72.02Ma)
21.34	top *Reinhardtites levis* (N, 70.14Ma)
22.86	base *Gansserina gansseri* (F, 72.97Ma), top *Tranolithus orionatus* (N, 71.01Ma), top *Uniplanarius trifidus* (N, 71.31Ma)
30.48	base *Arkhangelskiella maastrichtiana* (N, 74.51Ma)
36.58	base *Globotruncana aegyptiaca* (F, 74.00Ma)
82.30	top *Radotruncana calcarata* (F, 75.71Ma)
89.92	base *Radotruncana calcarata* (F, 76.18Ma)
91.44	base *Uniplanarius trifidus* (N, 76.82Ma),
100.58	top *Eiffellithus eximius* (N, 75.93Ma)
**134.11**	**base *Uniplanarius sissinghii* (N, 77.61Ma)**
167.64	base *Ceratolithoides aculeus* (N, 79.00Ma)
181.36	top *Lithastrinus grillii* (N, 79.73Ma)
**239.27**	**base *Broinsonia parca*** subsp. ***constricta* (N, 81.38Ma),** base *Bukryaster hayi* (N, 81.25Ma)
245.36	base *Broinsonia parca* subsp. *parca* (N, 81.43Ma)
251.46	presence of *Dicarinella asymetrica* (F, base at 86.67Ma)
**252.83**	**presence of *Arkhangelskiella cymbiformis* (N, base at 83.20Ma)**

Supplementary Table 2: Table of TEX_{86} data, showing sample depth, age, GDGT-indices[13,14], BIT index[45], MI index[17] and calculated SSTs based upon TEX_{86}^{L}, TEX_{86}^{H} and BAYSPAR[19] (Prior Mean: 30, Prior STD: 6) from the Shuqualak-Evans borehole. *This sample is assigned a minimum age of 66 Ma. See discussion in the text. ♯There is some uncertainty associated with the age of this sample due to slow sedimentation rates around the Santonian/Campanian boundary interval, but for the purposes of Figure 3 and Supplementary Figure 4 it is assigned an age minimum age of 82.87 based upon the age model shown in Supplementary Figure 2.

Depth (m)	Age (Ma)	Log TEX_{86}^{L} (GDGT index 1)	TEX_{86}^{L}-SST	TEX_{86}	Log TEX_{86}^{H} (GDGT index 2)	TEX_{86}^{H}-SST	TEX_{86}-SST (BAYSPAR)	BIT index	MI index
9.45	66.00*	-0.332	24.5	0.780	-0.108	31.2	32.1	0.098	0.186
10.97	66.43	-0.367	22.1	0.737	-0.132	29.5	29.5	0.097	0.197
12.80	67.30	-0.384	21.0	0.707	-0.150	28.3	27.7	0.076	0.219
13.72	67.74	-0.376	21.5	0.720	-0.143	28.8	28.5	0.088	0.215
15.24	68.46	-0.358	22.7	0.743	-0.129	29.8	29.9	0.099	0.204
16.76	69.18	-0.347	23.4	0.750	-0.125	30.1	30.3	0.102	0.199
18.29	69.91	-0.368	22.1	0.735	-0.134	29.5	29.4	0.080	0.201
19.81	70.63	-0.358	22.8	0.751	-0.124	30.1	30.3	0.077	0.200
21.34	71.35	-0.401	19.8	0.702	-0.154	28.1	27.4	0.090	0.216
22.86	72.08	-0.383	21.1	0.721	-0.142	28.9	28.5	0.092	0.215
25.91	73.52	-0.379	21.3	0.725	-0.140	29.1	28.8	0.118	0.202
30.48	73.89	-0.377	21.5	0.723	-0.141	29.0	28.7	0.053	0.225
33.53	74.00	-0.409	19.3	0.709	-0.149	28.4	27.8	0.128	0.213
39.62	74.22	-0.402	19.8	0.709	-0.149	28.4	27.9	0.098	0.226
45.72	74.44	-0.397	20.1	0.726	-0.139	29.1	28.9	0.146	0.216
51.82	74.66	-0.374	21.7	0.756	-0.121	30.3	30.6	0.115	0.193
59.44	74.93	-0.374	21.6	0.762	-0.118	30.5	30.9	0.116	0.197
62.48	75.04	-0.342	23.8	0.782	-0.107	31.3	32.2	0.147	0.203
68.58	75.26	-0.334	24.3	0.792	-0.101	31.7	32.8	0.119	0.184
74.68	75.48	-0.308	26.1	0.811	-0.091	32.4	33.9	0.138	0.177
82.30	75.75	-0.322	25.1	0.795	-0.100	31.8	32.9	0.111	0.181
86.87	75.91	-0.331	24.5	0.793	-0.101	31.7	32.8	0.123	0.168

92.96	76.13	-0.331	24.5	0.778	-0.109	31.1	32.0	0.094	0.178
99.06	76.35	-0.331	24.6	0.783	-0.106	31.3	32.2	0.100	0.179
102.11	76.46	-0.351	23.2	0.762	-0.118	30.5	31.0	0.101	0.181
108.20	76.68	-0.337	24.2	0.779	-0.109	31.2	32.0	0.125	0.184
115.82	76.95	-0.334	24.3	0.776	-0.110	31.1	31.8	0.108	0.175
120.40	77.12	-0.323	25.1	0.795	-0.100	31.8	33.0	0.099	0.169
129.54	77.45	-0.320	25.3	0.793	-0.101	31.7	32.8	0.102	0.169
132.59	77.55	-0.331	24.5	0.791	-0.102	31.6	32.7	0.115	0.176
137.16	77.72	-0.321	25.2	0.782	-0.107	31.3	32.2	0.049	0.203
144.78	77.99	-0.324	25.0	0.792	-0.101	31.7	32.8	0.108	0.184
150.88	78.21	-0.334	24.4	0.793	-0.101	31.7	32.8	0.105	0.189
156.97	78.43	-0.324	25.1	0.830	-0.081	33.1	35.0	0.083	0.159
163.07	78.65	-0.331	24.5	0.811	-0.091	32.4	33.9	0.076	0.161
167.64	78.81	-0.337	24.1	0.796	-0.099	31.8	33.0	0.042	0.178
172.21	78.98	-0.311	25.9	0.829	-0.081	33.0	35.0	0.064	0.151
181.36	79.31	-0.327	24.9	0.830	-0.081	33.1	35.0	0.084	0.146
187.45	79.52	-0.304	26.4	0.826	-0.083	32.9	34.8	0.048	0.167
193.55	79.74	-0.325	25.0	0.840	-0.076	33.4	35.6	0.062	0.140
202.69	80.07	-0.297	26.8	0.862	-0.064	34.2	36.9	0.072	0.132
210.31	80.35	-0.307	26.2	0.842	-0.075	33.5	35.7	0.054	0.146
217.93	80.62	-0.288	27.5	0.868	-0.061	34.4	37.3	0.064	0.136
227.08	80.95	-0.284	27.7	0.865	-0.063	34.3	37.1	0.058	0.138
234.70	81.22	-0.273	28.5	0.902	-0.045	35.5	39.3	0.064	0.109
239.27	81.38	-0.282	27.8	0.874	-0.058	34.6	37.6	0.077	0.135
245.36	81.60	-0.304	26.4	0.862	-0.064	34.2	36.9	0.070	0.146
251.46	82.87[♯]	-0.292	27.2	0.887	-0.052	35.0	38.4	0.059	0.129
